# TC1(C8orf4) Regulates Hematopoietic Stem/Progenitor Cells and Hematopoiesis

**DOI:** 10.1371/journal.pone.0100311

**Published:** 2014-06-17

**Authors:** Yusun Jung, Minsung Kim, Hyunsu Soh, Soyoung Lee, Jungtae Kim, Surim Park, Kyuyoung Song, Inchul Lee

**Affiliations:** 1 Asan Institute for Life Sciences, Asan Medical Center, University of Ulsan College of Medicine, Seoul, Korea; 2 Department of Biochemistry and Molecular Biology, Asan Medical Center, University of Ulsan College of Medicine, Seoul, Korea; 3 Department of Pathology, Asan Medical Center, University of Ulsan College of Medicine, Seoul, Korea; Emory University, United States of America

## Abstract

Hematopoiesis is a complex process requiring multiple regulators for hematopoietic stem/progenitor cells (HSPC) and differentiation to multi-lineage blood cells. TC1(C8orf4) is implicated in cancers, hematological malignancies and inflammatory activation. Here, we report that Tc1 regulates hematopoiesis in mice. Myeloid and lymphoid cells are increased markedly in peripheral blood of *Tc1*–deleted mice compared to wild type controls. Red blood cells are small-sized but increased in number. The bone marrow of *Tc1* ^−/−^ mice is normocellular histologically. However, Lin^−^Sca-1^+^c-Kit^+^ (LSK) cells are expanded in *Tc1* ^−/−^ mice compared to wild type controls. The expanded population mostly consists of CD150^−^CD48^+^ cells, suggesting the expansion of lineage-restricted hematopoietic progenitor cells. Colony forming units (CFU) are increased in *Tc1* ^−/−^ mice bone marrow cells compared to controls. In wild type mice bone marrow, Tc1 is expressed in a limited population of HSPC but not in differentiated cells. Major myeloid transcriptional regulators such as Pu.1 and Cebpα are not up-regulated in *Tc1* ^−/−^ mice bone marrow. Our findings indicate that TC1 is a novel hematopoietic regulator. The mechanisms of TC1-dependent HSPC regulation and lineage determination are unknown.

## Introduction

Hematopoiesis is a well orchestrated complex process under tight regulation. Numerous mature blood cells of multiple distinct lineage are produced everyday from small number of hematopoietic stem cells (HSC) (reviewed in [1]–[3]). The lineage fate is controlled in large part by distinct sets of transcriptional factors that may function independently or in combinations (reviewed in [4]–[6]). Bone marrow cytokines also play an important role in the hematopoietic regulation (reviewed in [7]). The regulation of hematopoietic stem/progenitor cells (HSPC) and hematopoiesis is one of the most extensively studied in biomedical science, including numerous animal studies *in vivo* [8]. However, our understanding of the complex regulation is still limited, and more regulators may wait to be identified.

*TC1*(*C8orf4*) is well conserved among vertebrates. It has been implicated in various cancers [9]–[13]. In stomach cancers, it has been implicated in poor prognosis of patients [12]. The copy number variation of *TC1* is associated with acute myeloid leukemia and other hematological malignancies [14], [15], suggesting a potential role in the hematopoietic regulation. In zebrafish embryos, two homologues are expressed in association with hematopoietic cells and blood vessels, respectively [16].

TC1 is a natively disordered protein [10], and undergoes rapid proteosomal degradation [17]. The expression is enhanced by pro-inflammatory cytokines, heat shock, and various cellular stresses [18], [19]. It enhances Wnt/β-catenin signaling in cancer cells and is associated with poor prognosis [12], [17]. It also induces inflammatory activation of endothelial cells enhancing classical NF-kB signaling [16]. It regulates the expression of pro-inflammatory downstream genes, suggesting a role of transcriptional regulator [12], [16], [17]. It has recently been implicated in Parkinson's disease in a genome-wide association study (GWAS), suggesting an aberrant regulation of innate immunity and/or blood vessels in brain [20].

To investigate the biological role *in vivo*, we have constructed a gene-targeted mouse line. Here, we report a novel role of Tc1 as a hematopoietic regulator.

## Materials and Methods

### Mice

*Tc1*-targeted mice were developed using a neomycinR inserted targeting construct transfected to the 129 strain ES cell. They have been backcrossed to C57BL/6 mice for 10 generations in specific pathogen-free (SPF) animal facility at the Asan Institute for Life Sciences, Seoul, Korea. All animal studies were performed with the approval by the Animal Care and Use Committee of the Asan Institute for Life Sciences following the experimental guidelines (Permit Number: 2012-12-054).

### Peripheral blood cell analysis

Peripheral blood samples were obtained from anesthetized mice by inferior vena cava puncture using a 26 gauge needle and 1 mL syringe containing heparin sodium (Sigma Aldrich, St. Louis, USA). Complete blood counts (CBC) and differential white blood cell (WBC) counts were done using an ADVIA 2120i Hematology System (Siemens Healthcare, Erlangen, Germany). Blood smears were stained with diffQuick (Merck, Darmstadt, Germany).

### Bone marrow cell separation

Bone marrow cells were flushed from femurs and tibias and filtrated through a cell strainer with 100-µm nylon mesh (BD Biosciences, San Jose, CA). Lineage-negative cells were obtained using a Lineage Cell Depletion Kit covering Ter119, Gr-1, CD11b, CD41, CD3e, and B220 (Miltenyi Biotec, Gladbach, Germany). Lin^−^Sca-1^+^ cells were sorted from Sca-1 stained Lin^−^ fraction using by FACSCalibur (BD Biosciences) flow cytometer as indicated.

### Colony-forming unit assay

Bone marrow cells obtained through flushing femurs and tibias using Iscove's Modified Dulbecco's Medium with 2% FBS (Life Technologies, Carlsbad, CA). Three independent experiments were done in duplicates and plated on dual-chamber slides. For CFU-GM, CFU-GEMM and BFU-E assay, 2×10^4^ cells were plated in 35-mm dish of MethoCult GF M3434 with recombinant mouse SCF, rmIL-3, recombinant human IL-6 and rhEpo. For each CFU-E assay, 2×10^5^ cells were plated with rhEpo, according to manufacturer's instructions (StemCell Technologies, Vancouver, BC, Canada). Colonies were blindly scored using microscope after 2 days of culture at 37°C with 5% CO_2_ and 95% humidity for CFU-E and at 7–12 days for CFU-GM, CFU-GEMM and BFU-E. Cells were harvested from plates with CFU colonies for flow cytometry. For repeated plating analysis, 1×10^4^ Lin^−^ cells were re-plated on MethoCult GF M3434 agar similarly up to 3 times.

### Flow cytometry

Single-cell suspension was prepared in ice-cold PBS containing 5% FBS, and 10^6^ cells were analyzed with 1µg antibody using FACSCantoII (BD Biosciences) flow cytometer. FITC-conjugated anti-CD11b, PE-conjugated F4/80 (BD Biosciences), FITC-conjugated Sca-1, PE-conjugated c-Kit (BD Biosciences), Alexa Fluor 647-conjugated Pu.1 (Cell Signaling Technology, Danvers, MA), APC-conjugated anti mouse CD150 and PerCP-conjugated anti CD48 antibodies (eBioscience Inc, San Diego, CA) were used as indicated. For Tc1 immunostaining, cells were fixed for 20 min at 4°C using Cytofix/Cytoperm kit (BD Biosciences). Anti-Tc1 antibody (Santa Cruz Biotechnology) and Alexa Fluor 594- or 488-conjugated anti-rabbit IgG secondary antibodies (Molecular Probes Inc, Eugene, OR) were applied sequentially after washing. FACSDiva software (BD Biosciences) and FlowJo (Tree Star) were used for data acquisition and analysis.

### Confocal immunofluorescence microscopy

1.2×10^5^ cells suspended in PBS were cytocentrifuged x1,000 rpm for 1 min onto glass slide. Slides were air-dried at room temperature and fixed in 100% methanol for 10 min at −20°C. Cells were immunostained using anti-Tc1 antibody (1∶50 dilution) for 3 h. After washing with PBS, Alexa Fluor 594-conjugated anti-rabbit IgG secondary antibody was applied in 1∶200 dilution for 1 h. Nuclear staining was done using Hoechst 33342 (Sigma-Aldrich). Cells were viewed using an inverted zeiss LSM 710 confocal microscopy (Carl Zeiss, Thornwood, NY).

### Cytokine array and western blotting

For cytokine array, flushed bone marrow cells were gently washed with serum-free RPMI 1640 (Gibco, Carlsbad, CA) media for 10 min at room temperature, and the media was recovered by centrifugation at 300 g for 5 min. 250 µg of total proteins measured by Bradford assay were applied to a mouse cytokine array (Raybiotech, Norcross, GA), as described previously (16). For western blotting, total tissue samples were solubilized in lysis buffer, and loaded, 20 µg per lane, on 12% SDS-PAGE. Proteins were blotted onto nitrocellulose membrane and probed using anti-TC1 antibody. Anti-β-actin antiserum was used for loading control (Sigma-Aldrich).

### Quantitative RT-PCR

Total RNA was extracted using Trizol reagent (Invitrogen, Carlsbad, CA), and cDNA was synthesized using Premium reverse transcriptase (Thermo Scientific, Marietta, OH). qPCR was performed using a continuous fluorescence detecting thermal cycler ABI PRISM 7000 Sequence Detection System (ABI, Foster city, CA), and a SYBR Green real-time PCR master mix (Toyobo, Osaka, Japan). Measurements were done in triplicate using β-actin as endogenous control. PCR primers are summarized in Table 1.

**Table 1:** Quantitative PCR primers.

	Genes	sequences (5′ to 3′)		Amplicon
		Forward	Reverse	(bp)
**Human**	**ACTB**	GGCACCCAGCACAATGAAG	GCCGATCCACACGGAGTACT	67
	**TC1**	CAAGCCATCATCATGTCCAC	GTTGCCCACGGCTTTCTTAC	86
	**PU.1**	GACACGGATCTATACCAACGCC	CCGTGAAGTTGTTCTCGGCGAA	145
	**TREM-1**	AAGGAGCCTCACATGCTGTT	CACAGTTCTGGGGCTGGTAT	160
**Mouse**	**Actb**	GATCATTGCTCCTCCTGAGC	ACATCTGCTGGAAGGTGGAC	83
	**Ccnd-1**	CCCAACAACTTCCTCTCCT	TCCAGAAGGGCTTCAATCTG	110
	**Cebpα**	TTACAACAGGCCAGGTTT	CTCTGGGATGGATCGATT	232
	**Flt3l**	CGCTGGATAGAGCAACTGAAGA	TGTTGGTCTGGACGAATCGCAG	138
	**Gata-1**	ACTGTGGAGCAACGGCTA	TTCCTCGTCTGGATTCCA	301
	**G-CSF**	ATGCCGAAGGCTTCCCTG	AGGAGACCTTGGTAGAGG	98
	**GM-CSF**	AACCTCCTGGATGACATG	AAATTGCCCCGTAGACCC	133
	**Tc1**	ACCAGCATGTCCTCGTCTCT	ATGTTGCCCACAGCTTTCTT	89
	**Klf4**	CTATGCAGGCTGTGGCAAAACC	TTGCGGTAGTGCCTGGTCAGTT	150
	**M-CSF**	GCCTCCTGTTCTACAAGT	ACTGGCAGTTCCACCTGT	125
	**Myc**	AGTGCTGCATGAGGAGACAC	GGTTTGCCTCTTCTCCACAG	103
	**Pou5f1**	CAGCAGATCACTCACATCGCC	GCCTCATACTCTTCTCGTTGGG	128
	**Pu.1**	TACCAACGTCCAATGCATGA	GCTGGGGACAAGGTTTGATA	302
	**Sox2**	AACGGCAGCTACAGCATGATGC	CGAGCTGGTCATGGAGTTGTAC	138
	**Trem-1**	TTTCCTCTCCTGGTCTTGGA	TCATCCAAATGTCCTCTTTGTG	155

### *TC1*-transfected HL-60 cells

HL-60 cells were cultured in RPMI media, supplemented with 10% FBS and 100U/ml penicillin and 100 µg/ml streptomycin (Gibco), at 37°C in humidified atmosphere with 5% CO_2_. 1×10^7^ of HL-60 cells at passages 4–8 were harvested in the exponential growth phase and resuspended in Gene Pulser Electroporation buffer (Bio-Rad, Hercules, CA). Cells were mixed with *TC1-*pCAGGS mammalian expression vector (Addgene, Cambridge, MA) or vectors only, and pulsed at 175V, 1000 µF using 4 mm cuvette by Gene Pulser Xcell Electroporation Systems (Bio-Rad).

### Statistical Methods

All measurements are presented as the mean ± s.d. Statistical analyses were performed by two-tailed *t* -tests by SPSS version 20 (SPSS Inc., Chicago, IL).

## Results

### Myeloid and lymphoid hyperplasia in *Tc1* ^−/−^ mice blood

**Figure 1:**
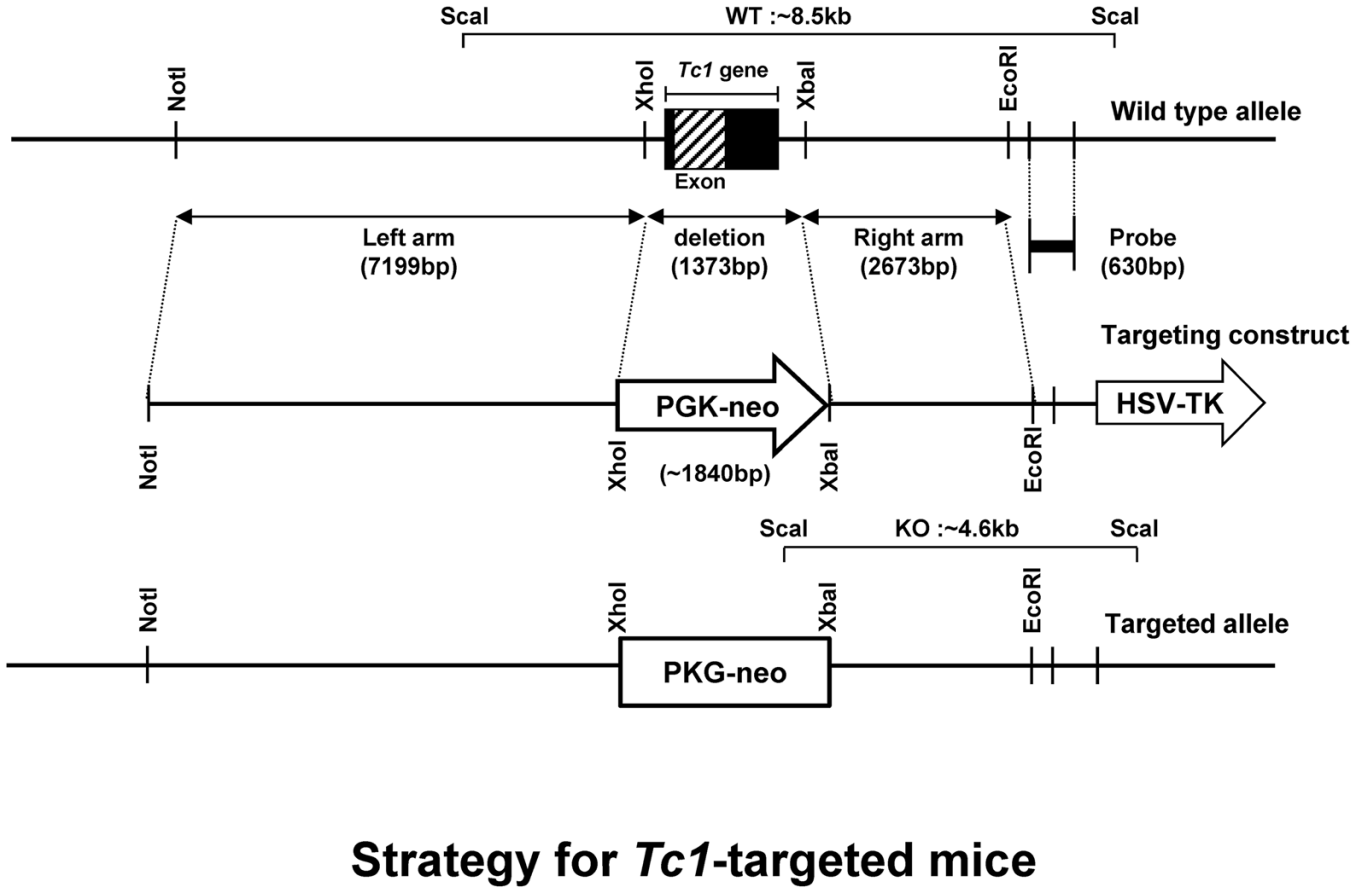
*Tc1*-targeting strategy. Restriction maps of wild type allele, targeting construct and targeted allele. The probe used for Southern blot analysis is indicated. The top line represents the structure and partial restriction map from wild-type allele of *Tc1*. The middle and low lines depict the targeting construct and predicted structure of targeted allele, respectively.

*Tc1*-targeted mice were generated (Figure 1 and Figure S1), and were back-crossed to C57BL/6 mice for 10 generations. Systemic deletion in homozygotes was confirmed by RT-PCR (Figure S2) and western blotting (data not shown). *Tc1*-deleted homozygotes have thrived under the SPF environment. Interestingly, the hematological profile changed remarkably.

**Figure 2:**
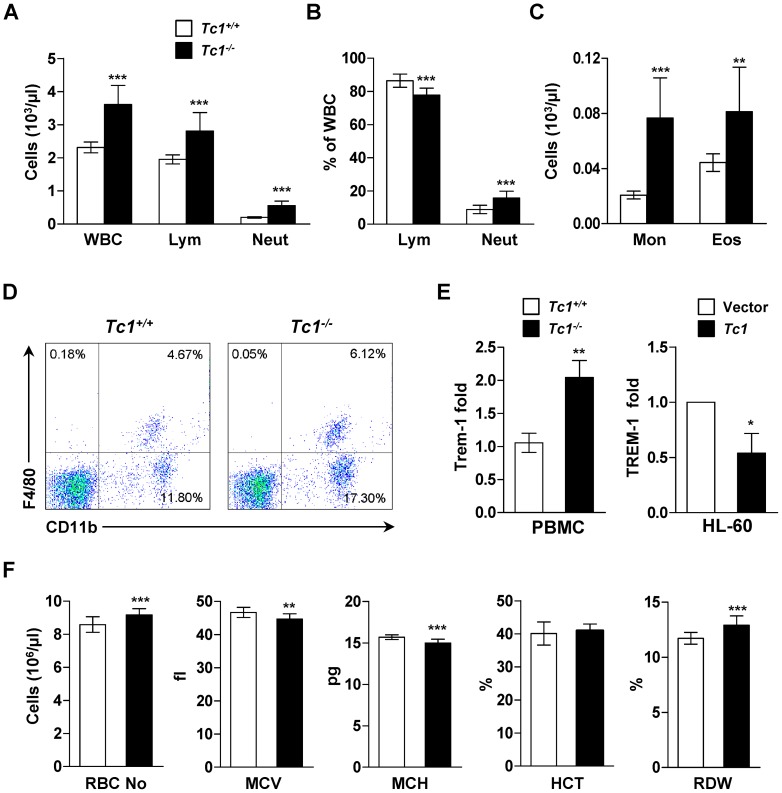
Enhanced hematopoiesis in *Tc1* ^−/−^ mice. (A) Numbers of white blood cells (WBC), lymphocytes (Lym) and neutrophils (Neut). Data for A, B, C, and F represent mean ± s.d. of 14 male, 7 to 9 week-old *Tc1* ^−/−^ mice and, 14 sex- and age-matched control mice over 5 independent experiments. (B) The percentage of lymphocytes and neutrophils per total WBC. (C) Numbers of monocytes (Mon) and eosinophils (Eos). (D) Representative flow cytometric analysis of peripheral blood mononuclear cells (PBMC) for Cd11b and F4/80 expression in wild type and *Tc1* ^−/−^ mice. (E) Left: qPCR analysis of Trem-1 expression in PBMC. Data represent mean ± s.d. of 6 male, 8 week-old *Tc1* ^−/−^ mice, and 6 sex-, and age-matched control mice over 3 independent experiments. Right: qPCR analysis of TREM-1 expression in *TC1*-transfected HL-60 cells and vector-transfected control. Data of expression fold difference represent mean ± s.d. of 3 independent experiments. (F) The RBC number, mean corpuscular volume (MCV), mean corpuscular hemoglobin (MCH), hematocrit (HCT), and red cell distribution width (RDW) in peripheral blood from control and *Tc1*-deleted mice. *p<0.05; **p<0.01; ***p<0.001.

Peripheral blood total WBC increased in young adult *Tc1* ^−/−^ mice compared to age-, and sex-matched wild type controls by 56.3% in average (*p*<0.001, Figure 2A). Neutrophils increased predominantly, 2.8 times of controls in average. Lymphocytes also increased by 44.1% in average. However, the percentage of lymphocytes in total WBC was rather decreased by 11.1% (*p*<0.001, Figure 2B), reflecting steeper increase of myeloid cells including neutrophils, monocytes, and eosinophils (Figure 2A, C). The expression of Cd11b and F4/80, myeloid and macrophage/monocyte markers respectively, increased in blood mononuclear cells of *Tc1* ^−/−^ mice compared to controls by flow cytometry (Figure 2D).

We then investigated whether WBCs of *Tc1* ^−/−^ mice were functionally active. Trem-1 is selectively expressed on activated neutrophils and monocytes to amplify the inflammatory response [21]. Trem-1 was up-regulated in blood mononuclear cells of *Tc1* ^−/−^ mice compared to controls by qPCR analysis (Figure 2E, left), suggesting that they were no less active than the wild type counterparts. To investigate a role of TC1 on the TREM-1 expression, human promyelocytic leukemia cell line HL-60 was transiently transfected with *TC1* using electroporation. TREM-1 was down-regulated in *TC1*-transfected HL-60 cells compared to vector-transfected control (Figure 2E, right), suggesting a potential role of TC1 in regulating myeloid cell activity.

### Increased number of small-sized RBCs in *Tc1* ^−/−^ mice

Red blood cells (RBC) also increased in *Tc1* ^−/−^ mice by 6.86% in average compared to controls (*p*<0.001, Figure 2F). However, the mean corpuscular volume (MCV) and mean corpuscular hemoglobin (MCH) decreased significantly, indicating that RBCs of *Tc1* ^−/−^ mice were smaller than controls. The hematocrit (HCT; Figure 2F) and mean corpuscular hemoglobin concentration (MCHC; data not shown) remained unchanged. No abnormal RBC was shown on blood smear (data not shown). The red cell distribution width (RDW), reflecting the RBC size variation, was higher in *Tc1* ^−/−^ mice than controls (Figure 2F, right).

### Expansion of LSK cells in *Tc1* ^−/−^ mice

**Figure 3:**
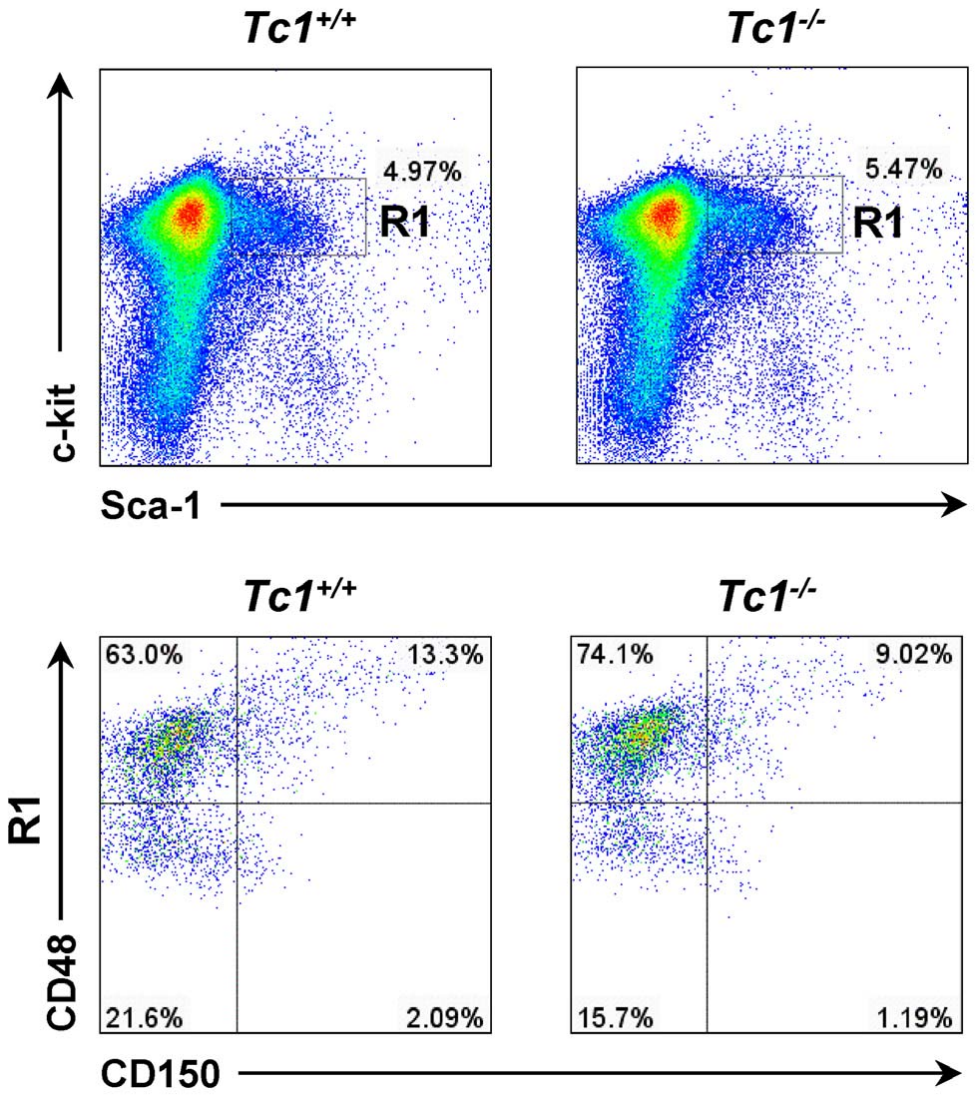
Expansion of LSK cells in *Tc1^−/−^* mouse bone marrow. Representative flow cytometric analysis for Lin^−^ bone marrow cells for Sca-1^+^c-Kit^+^ cells from *Tc1^−/−^* mice and matched control mice. Profiles for CD150 and CD48 co-expression in the gated LSK cells (R1) are shown, respectively.

The bone marrow of *Tc1* ^−/−^ mice showed normal cellularity, composition, and bone development (not shown). The lineage-negative bone marrow cells were analyzed using flow cytometry. Lin^−^Sca-1^+^c-Kit^+^ (LSK) cells were expanded in *Tc1* ^−/−^ mice bone marrow compared to controls (Figure 3), suggesting a role of Tc1 in the HSPC regulation. LSK cells were gated and further analyzed for CD150/Cd48 profiling [22], [23]. CD150^−^CD48^+^ subpopulations expanded evidently compared to wild type control (Figure 3), suggesting the expansion of lineage-restricted progenitor cells. The relative proportions of CD150^+^CD48^−^, CD150^+^CD48^+^, and CD150^−^CD48^−^ subpopulations appeared to be reduced in LSK cells of *Tc1* ^−/−^ mice compared to controls. However, the numbers of HSC or multipotent progenitor cells may not differ significantly considering the expansion of total Lin^−^ cells in *Tc1* ^−/−^ mice.

### Tc1 expression in primitive hematopoietic cells

**Figure 4:**
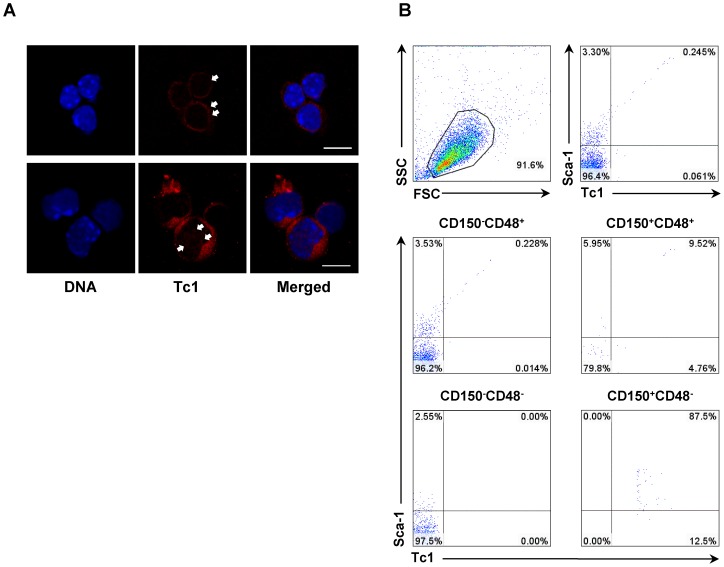
Tc1 expression in bone marrow cells. (A) Confocal microscopic images of Tc1-positive cells in wild type mice bone marrow. DNA staining is done using Hoechst 33342. Relatively weak cytoplasmic staining (arrows) is present in HSC-like primitive cells (upper panel). Large progenitor cells show strong cytoplasmic staining and faint nuclear staining (arrows) (lower panel). Scale bars represent 10 µm. (B) Representative flow cytometric analysis for Tc1 and Sca-1 in total Lin^−^ bone marrow cells (upper panels), and gated cells according to CD150/CD48 profiles (lower panels).

The expression of Tc1 in wild type C57BL/6 mouse bone marrow was investigated. Previously, TC1 has been reported to express at low level in bone marrow of humans and zebrafish embryos [9], [16], while the expression in peripheral blood is too low to be detected by RNA blotting [9], [17]. Upon immunofluorescence microscopy, Tc1 was expressed in small numbers of primitive hematopoietic cells. HSC-like cells with round nuclei of 6–8 µM in diameter and scant cytoplasm showed relatively weak cytoplasmic granular staining (Figure 4A, upper panel). Large progenitor-like cells often showed strong cytoplasmic and faint nuclear staining (Figure 4A, lower panel). The nuclear translocation of TC1 upon cellular stimuli was described, suggesting a role in the regulation of downstream gene expression [19]. Cells with ring-shaped nuclei were also immunostained for Tc1 (Figure S3A, B). Myeloid cells with ring-shaped nuclei were reported in mouse but not in humans normally [24]. Differentiated myeloid cells were not immunostained (Figure S3B, C).

Tc1 expression in lineage-negative bone marrow cells was investigated using flow cytometry. Tc1-expressing cells were infrequent, much less than 1% of total Lin^−^ cells (Figure 4B). Tc1^+^ cells mostly co-expressed Sca-1. Tc1 expression was further analyzed in gated cells according to CD150/CD48 profiles. Most CD150^+^CD48^−^ cells were positive for Tc1, supporting the expresssion of Tc1 by HSCs (Figure 4B) [22], [23]. Tc1 was also expressed in significant proportions of CD150^−^CD48^+^ and CD150^+^CD48^+^ cells, corresponding to lineage-restricted progenitor cells [22], [23].

### Increased colony forming units in *Tc1* ^−/−^ mice bone marrow

**Figure 5:**
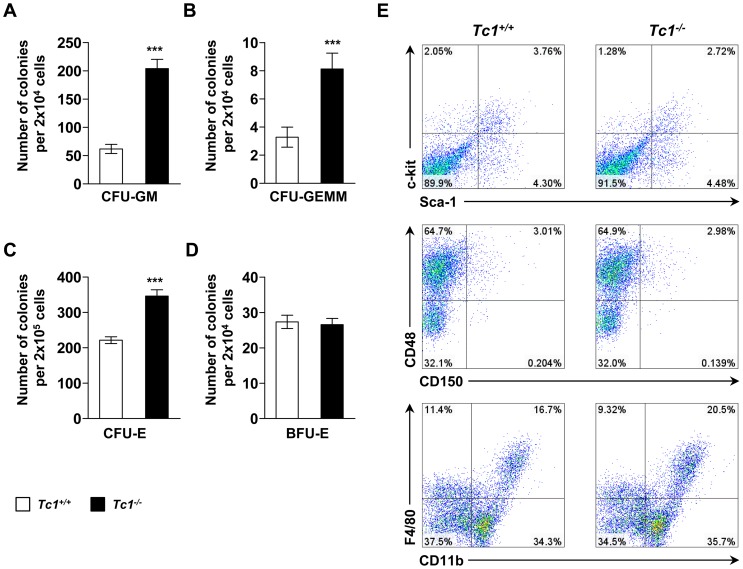
*In vitro* colony-forming unit (CFU) assay. Data represent mean ± s.d. of colony numbers per plated cells as indicated of 8 male, 8 week-old *Tc1* ^−/−^ mice, and 8 sex- and age-matched control mice over 3 independent experiments. (A) CFU-granulocyte-macrophage (CFU-GM). (B) CFU-granulocyte-erythroid-monocyte-megakaryocyte (CFU-GEMM). (C) CFU-erythroid (CFU-E). (D) burst-forming unit-erythroid (BFU-E). ***P<0.001. (E) Representative flow cytometric analysis of recovered cells from the CFU plates of *Tc1^−/−^* mice and matched control mice.

We investigated the hematopoietic activity of lineage-negative cells from *Tc1* ^−/−^ and wild type mice bone marrow using colony forming unit (CFU) assay. CFU-granulocyte-macrophage(GM) and CFU-granulocyte-erythroid-monocyte-megakaryocyte(GEMM) increased in *Tc1* ^−/−^ mice compared to controls (Figure 5A, B and Figure S4). CFU-E also increased significantly in *Tc1* ^−/−^ mice (Figure 5C). Burst-forming unit(BFU)-E, which represent erythroid hematopoietic activity at upper hierarchy, did not increase compared to controls (Figure 5D). Total CFU-GM containing cells were recovered and analyzed using flow cytometry. The profiles of Sca-1, c-Kit, CD150, and CD48 expression were similar between *Tc1* ^−/−^ and wild type mice (Figure 5E). CD11b and F4/80 expressing cells appeared to be mildly increased in *Tc1* ^−/−^ colonies compared to controls, suggesting up-regulated potential for myelopoiesis.

To investigate the self-renewal activity, we serially re-plated cells applying the same number of cells to each plate similarly. The colony forming activities were maintained on repeated plating in cells from *Tc1* ^−/−^ and wild type mice (Figure S5). Colonies from *Tc1* ^−/−^ mice bone marrow appeared to be less than wild type controls upon re-plating, probably reflecting relatively reduced proportions of HSC compared to expanded myeloid progenitors in the given number of repeatedly plated cells.

### Unknown mechanism for lineage regulation

**Figure 6:**
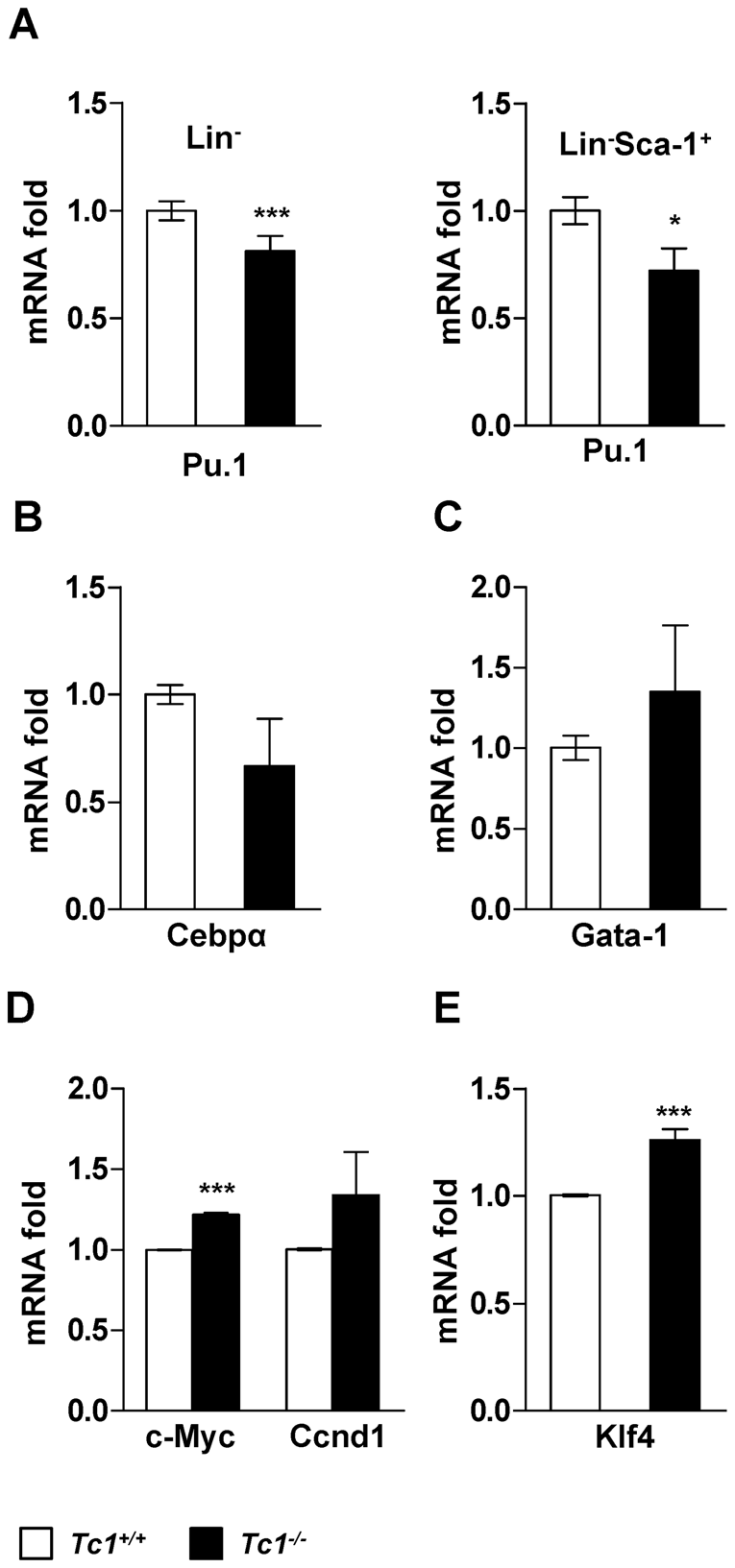
Hematopoietic lineage- and pluripotent stem cell-regulators. (A) qPCR analysis for Pu.1 expression in total Lin^−^ cells (left) or sorted Lin^−^Sca-1^+^ cell fraction (right) of *Tc1^−/−^* and wild type mice bone marrow. Data represent mean ± s.d. of 6 male, 8 week-old *Tc1* ^−/−^ mice, and 6 sex- and age-matched control mice over 3 independent experiments for the total Lin^−^ cell assay, and 10 male, 8 to 9 week-old *Tc1* ^−/−^ mice, and 10 sex- and age-matched control mice over 2 independent experiments for the sorted-Lin^−^Sca-1^+^ fraction assay, respectively. (B–E) qPCR analysis for Cebpα (B), Gata-1 (C), c-Myc and Ccnd1 (D), and Klf4 (E) expression in Lin^−^ cells. Data represent mean ± s.d. of 6 male, 9 week-old *Tc1* ^−/−^ mice, and 6 sex- and age-matched control mice over 3 independent experiments. **p<0.01; ***p<0.001.

Our data together supported a biological role of Tc1 in the regulation of HSPC *in vivo*. However, it was not clear how the relatively myeloid- and lymphoid-prone lineage fate was determined in *Tc1* ^−/−^ mice. We first investigated the expression of major transcriptional regulators for myelopoiesis [25]. Upon flow cytometry, the expression of Pu.1 was not increased in LSK cells of *Tc1* ^−/−^ mice compared to wild type controls (data not shown). By qPCR analysis, Pu.1 was down-regulated significantly in total Lin^−^ from *Tc1* ^−/−^ mice bone marrow compared to controls (Figure 6A). Pu.1 expression was also down-regulated in sorted Lin^−^Sca-1^+^ cells of *Tc1* ^−/−^ mice. To investigate a potential TC1-dependent regulation of PU.1, HL-60 cells were transfected using *TC1*. No significant change in the PU.1 expression was shown in *TC1*-transfected HL-60 cells compared to vector-transfected control (data not shown). Cebpα, another major regulator of myelopoiesis, was also down-regulated marginally (*p*<0.07) in Lin^−^ cells of *Tc1* ^−/−^ mice compared to controls (Figure 6B). Gata-1, a major regulator for erythroid cell fate, appeared to be up–regulated in *Tc1* ^−/−^ mice mildly, but the difference was not statistically significant (Figure 6C).

Hematopoietic cell lineages may also be regulated by cytokines [26], [27]. The expression of bone marrow cytokines was investigated using a cytokine array. No differentially expressed cytokine was detected in *Tc1* ^−/−^ mice compared to controls (Figure S6). G-CSF and GM-CSF were not expressed enough to be detected. Upon qPCR of total bone marrow cells, the expression of G-CSF, GM-CSF, M-CSF, and Flt3l did not change in *Tc1* ^−/−^ mice compared to controls (Figure S7).

Wnt signaling is normally required for the regulation of HSPC [28]. Using qPCR, we investigated the expression of c-Myc and Cyclin D1, typical downstream genes of classical Wnt signaling. c-Myc was up-regulated significantly in Lin^−^ cells of *Tc1* ^−/−^ mice compared to controls (Figure 6D). Cyclin D1 appeared to be up–regulated mildly, but the difference was not statistically significant. c-Myc is one of the transcriptional regulators implicated in the regulation of pluripotent stem cells [29]. We investigated other regulators implicated in pluripotential stem cell induction including Klf4, Sox2, and Oct4. Klf4 was also up-regulated in Lin^−^ bone marrow cells of *Tc1* ^−/−^ mice compared to controls (Figure 6E). Sox2 and Oct4 were not detected upon repeated qPCR analysis (data not shown).

## Discussion

*Tc1*-deleted mice show increased blood cells and enhanced hematopoietic activity. LSK cells are expanded *in vivo* and CFUs are increased *in vitro*, supporting the role of Tc1 as a novel hematopoietic regulator. CD150^−^CD48^+^ cells are increased in gated LSK cells, supporting that lineage-restricted hematopoietic progenitor cells are expanded preferentially. Tc1 is expressed in a small population of hematopoietic cells including HSCs and lineage-restricted progenitor cells in wild type C57BL/6 mice bone marrow. Our data together supported a role of TC1 in negative hematopoietic regulation.

The hematopoietic activity is enhanced overall in *Tc1* ^−/−^ mice. Myeloid cells are expanded particularly, suggesting a potential role of Tc1 in the lineage fate determination. The mechanism of the skewed lineage regulation is not known. Myelopoietic transcriptional regulators Pu.1 and Cebpα are not up-regulated in hematopoietic cells from *Tc1* ^−/−^ mice compared to controls. Bone marrow cytokines such as G-CSF, GM-CSF, M-CSF, and Flt3l are not up-regulated, either. Further investigations are required for other transcriptional regulators, cytokines, and/or *in vivo* hematopoietic niche regulation.

Interestingly, RBCs are also expanded in number but small-sized in *Tc1*-deleted mice blood. The size of RBC may be influenced by environmental and genetic factors. RBCs of *Phyllotis xanthopygus rupestris*, a small Andean mammal, become significantly smaller during the winter whereas the hematocrit and total hemoglobin are not down-regulated [30]. The biological significance of small-sized RBCs is not clear. In humans, an ethnic difference in the RBC size has been reported in healthy individuals without iron deficiency anemia [31]. Genetic determinants for RBC parameters have been sought for extensively using GWAS [32], [33]. As far as we are aware of, *Tc1* is the first gene reported for definite RBC size determination. RBCs of *Tc1*-deleted mice also show increased size variation, RDW. Increased RDW is associated with cardiovascular and other adult diseases [34]. However, it is unknown whether it might be the cause or epiphenomenon of underlying diseases [35].

TC1 has been implicated in the inflammatory activation of endothelial cells [16]. In mouse bone marrow, Tc1 was expressed by some myeloid cells with ring-shaped nuclei, which have been associated with myeloid-derived suppressor cells (MDSC) in mice [36]. TC1-dependent TREM-1 regulation in HL-60 cells suggests a potential role of TC1 in the regulation of leukocyte activity. Together, our data suggest that TC1 may have complex biological roles for systemic regulation of hematopoiesis, innate immunity, and stem cells in vertebrates.

## Supporting Information

Figure S1 **Screening of** ***Tc1*** **-targeted mice by Southern blotting.** Genotyping of offspring from *Tc1^+/−^* × *Tc1^+/−^* using mouse tail DNA digested with ScaI. The 8.5- and 4.6-kilobase bands represent wild-type and mutant *Tc1* alleles, respectively.
(TIF)

Figure S2 **RT-PCR for Tc1 expression in total and lineage-negative bone marrow cells of wild type and Tc1-KO mice.**
(TIF)

Figure S3 **Confocal microscopic image of representative Tc1-expressing cells in bone marrow of wild type mice.** Scale bars  = 10 µm.
(TIF)

Figure S4 **Representative CFUs from** ***Tc1*** **^−/−^ and wild type mice bone marrow.** Bone marrow cells were cultured in methlyl cellulose plates as described, and colonies were scored blindly using inverted microscope.
(TIF)

Figure S5 **Figure S5: CFU-GM on repeated plating from T** ***c1*** **^−/−^ and wild type mice cells.** The same numbers of total cells from the primary CFUs were re-plated repeatedly. Data represent mean ± s.d. of 4 independent experiments. *p<0.05.
(TIF)

Figure S6 **Mouse cytokine array assay using bone marrow flushed serum-free media from** ***Tc1*** **^−/−^ and wild type mice.** The positions for G-CSF/GM-CSF (upper) and TIMP-1 (lower) are indicated in red squares. Positive and negative controls are indicated.
(TIF)

Figure S7 **qPCR analysis of total bone marrow cells for G-CSF, GM-CSF, M-CSF, and Flt3l.** Data represent mean ± s.d. of 6 male, 9 week-old *Tc1* ^−/−^ mice, and 6 sex- and age-matched control mice over 3 independent experiments.
(TIF)
