# Supplementary figures and images for: TC1(C8orf4) Regulates Hematopoietic Stem/Progenitor Cells and Hematopoiesis
Source: PLoS One. 2014 Jun 17;9(6):e100311. doi: 10.1371/journal.pone.0100311 (PMC4061086; doi:10.1371/journal.pone.0100311)

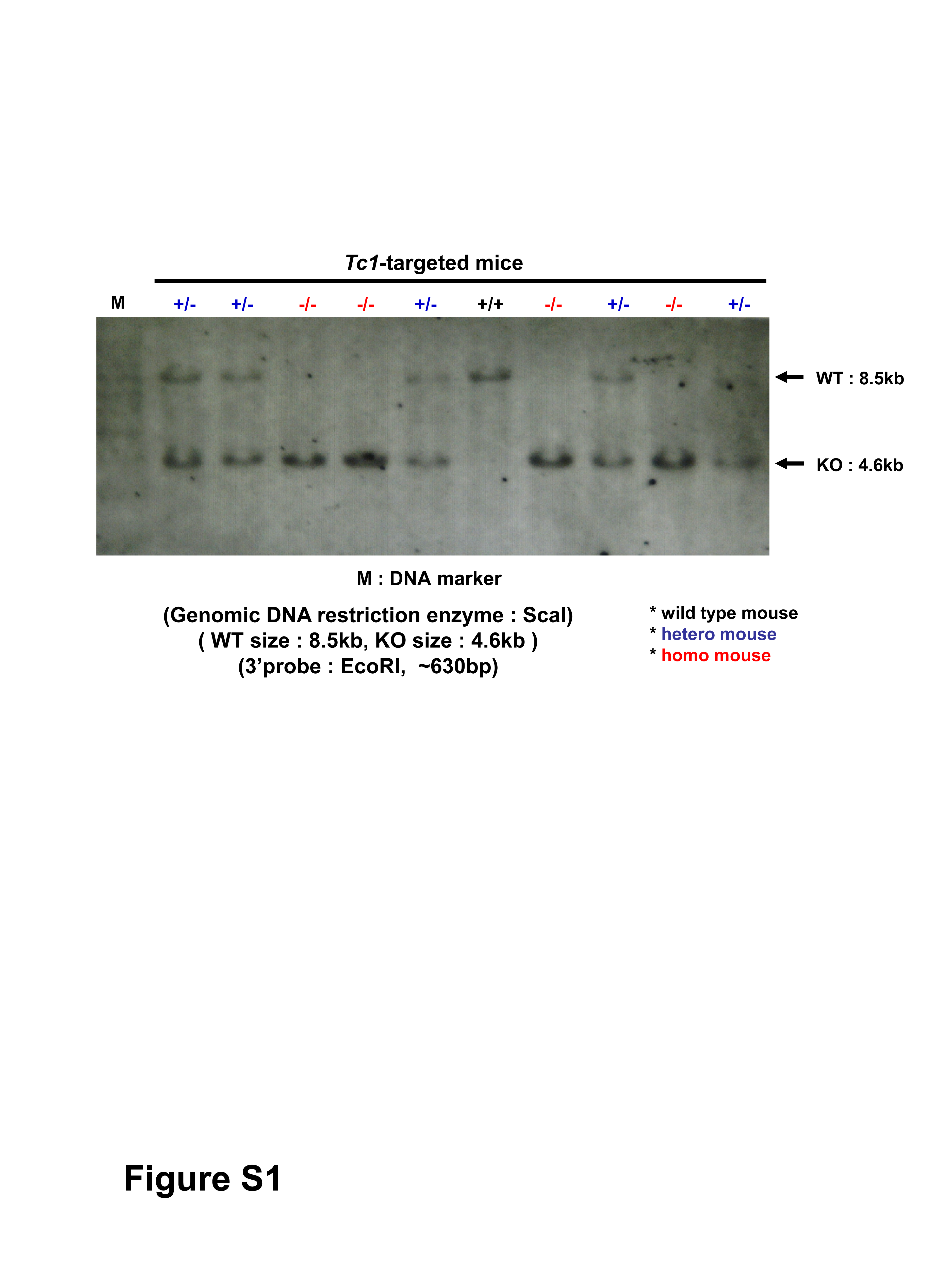

Supplement: Figure S1 — Screening of Tc1 -targeted mice by Southern blotting. Genotyping of offspring from Tc1+/− × Tc1+/− using mouse tail DNA digested with ScaI. The 8.5- and 4.6-kilobase bands represent wild-type and mutant Tc1 alleles, respectively. (TIF) [file pone.0100311.s001.tif]

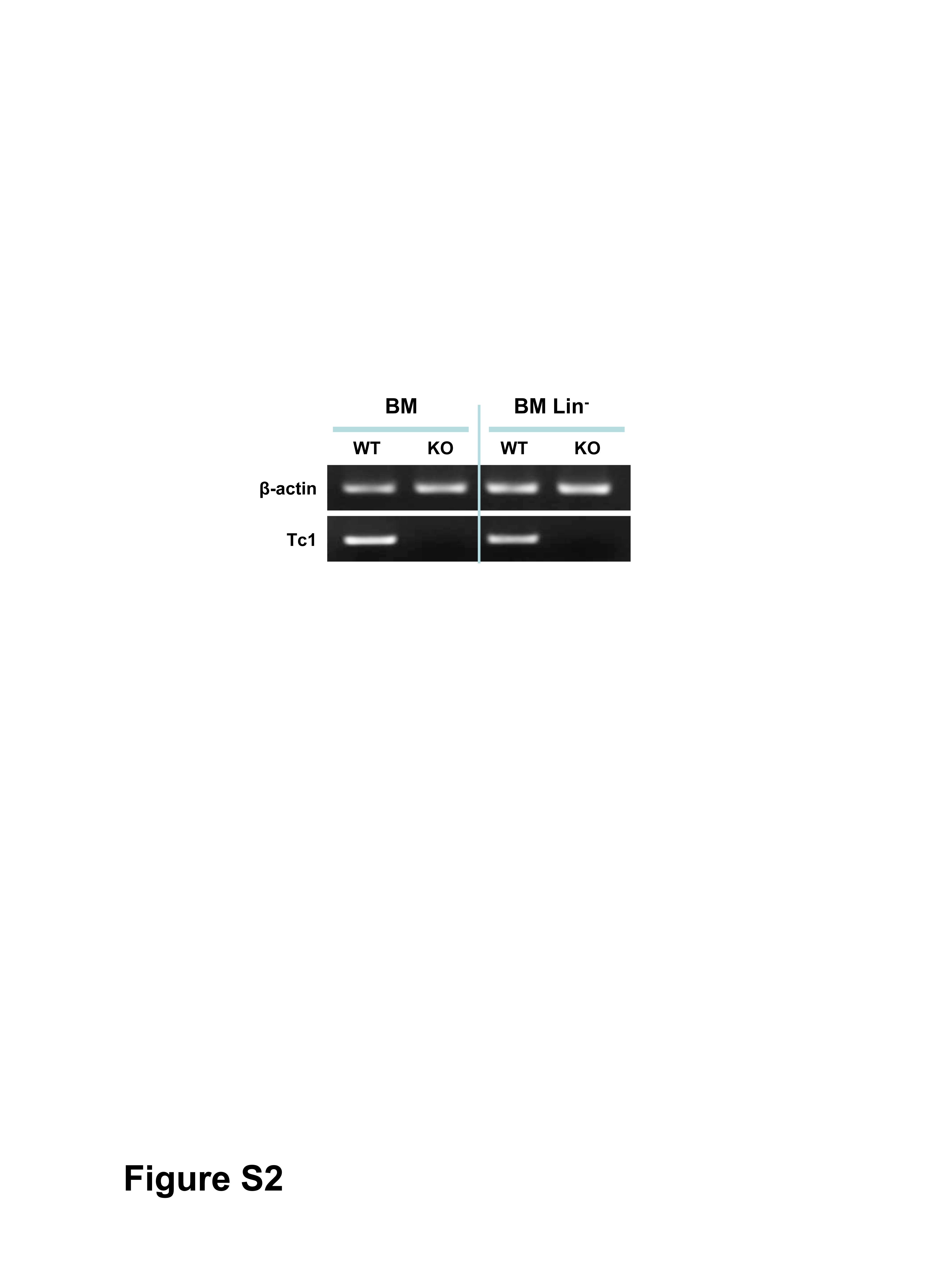

Supplement: Figure S2 — RT-PCR for Tc1 expression in total and lineage-negative bone marrow cells of wild type and Tc1-KO mice. (TIF) [file pone.0100311.s002.tif]

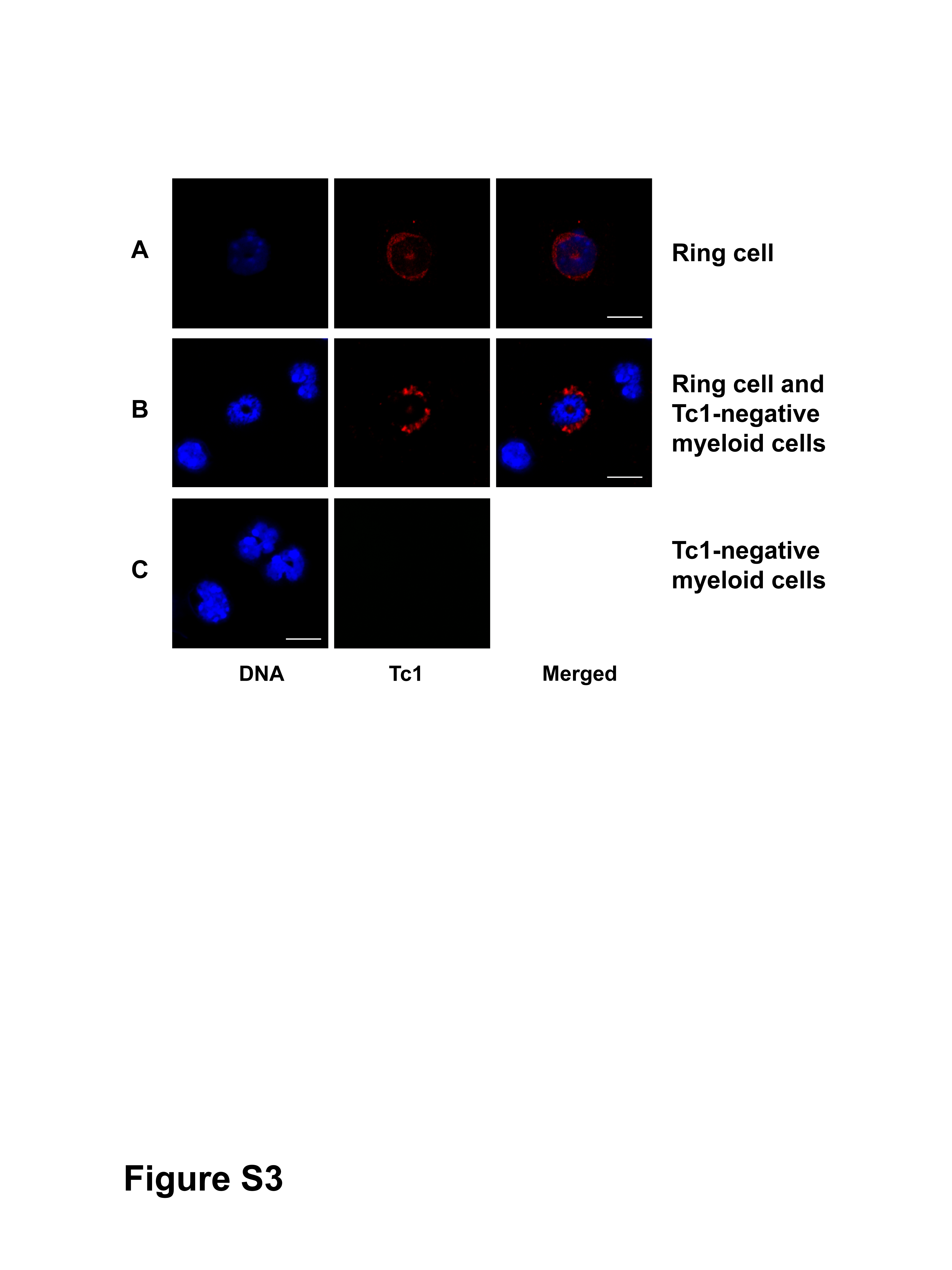

Supplement: Figure S3 — Confocal microscopic image of representative Tc1-expressing cells in bone marrow of wild type mice. Scale bars = 10 µm. (TIF) [file pone.0100311.s003.tif]

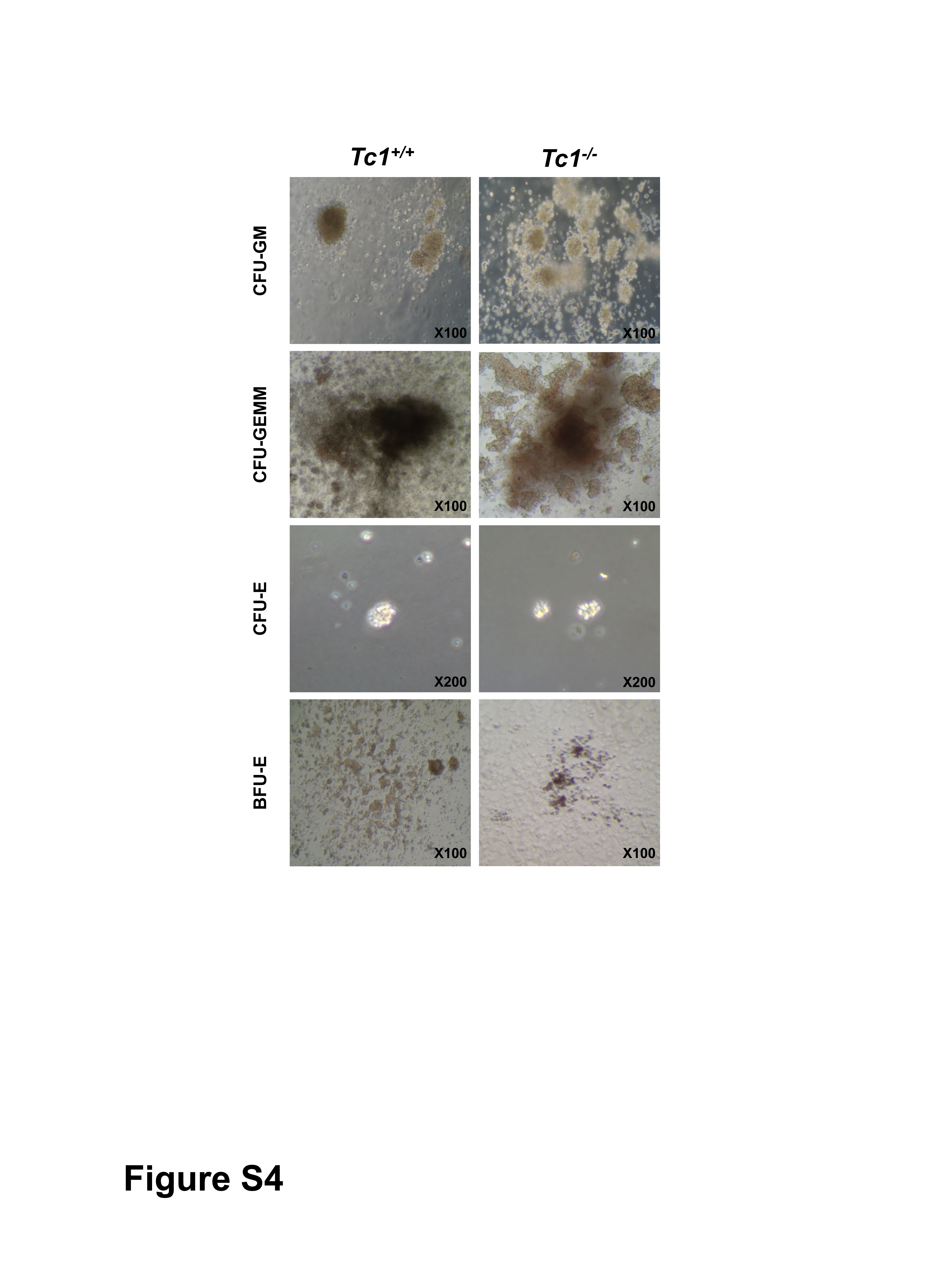

Supplement: Figure S4 — Representative CFUs from Tc1 −/− and wild type mice bone marrow. Bone marrow cells were cultured in methlyl cellulose plates as described, and colonies were scored blindly using inverted microscope. (TIF) [file pone.0100311.s004.tif]

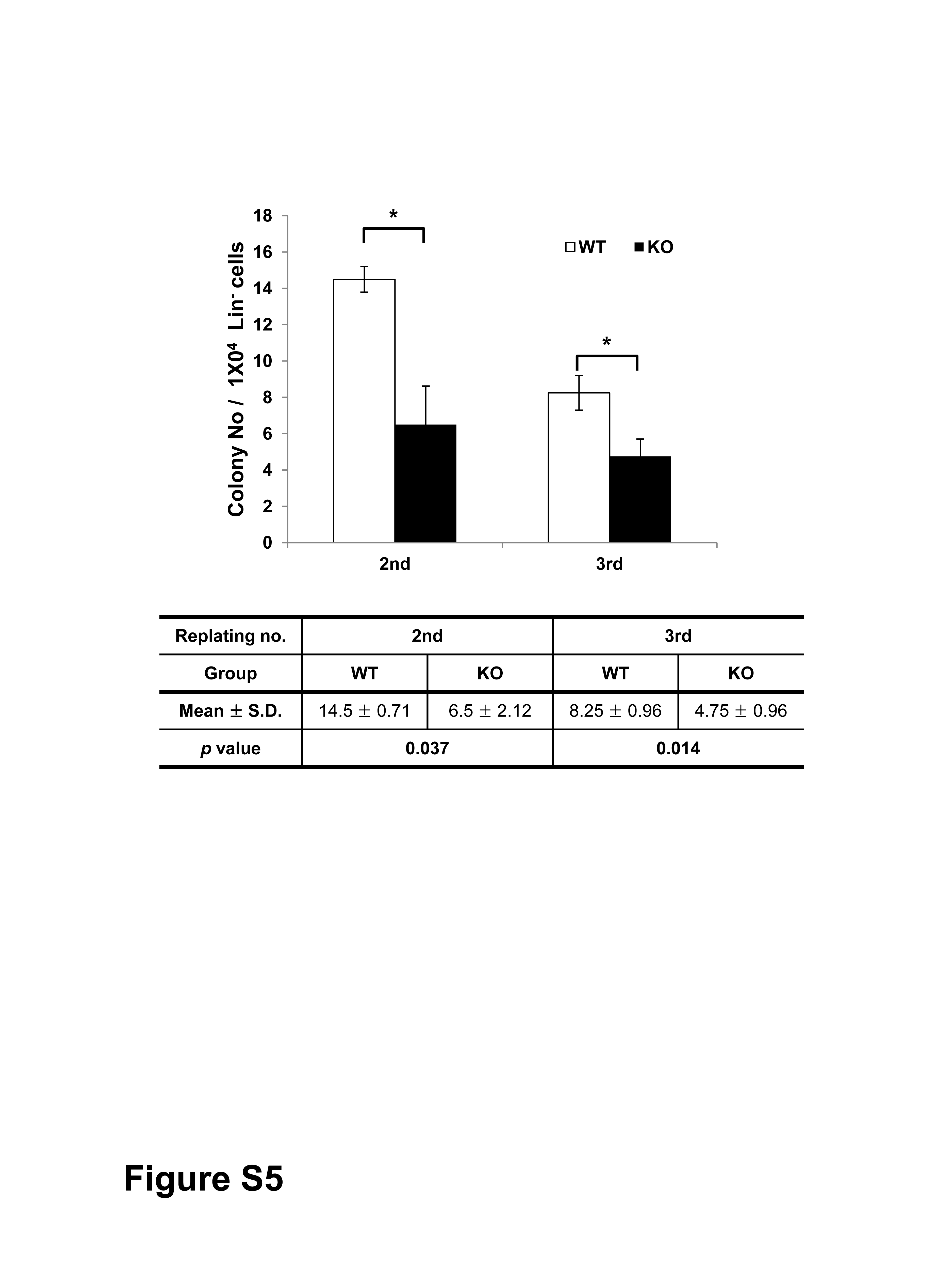

Supplement: Figure S5 — CFU-GM on repeated plating from T c1 −/− and wild type mice cells. The same numbers of total cells from the primary CFUs were re-plated repeatedly. Data represent mean ± s.d. of 4 independent experiments. *p<0.05. (TIF) [file pone.0100311.s005.tif]

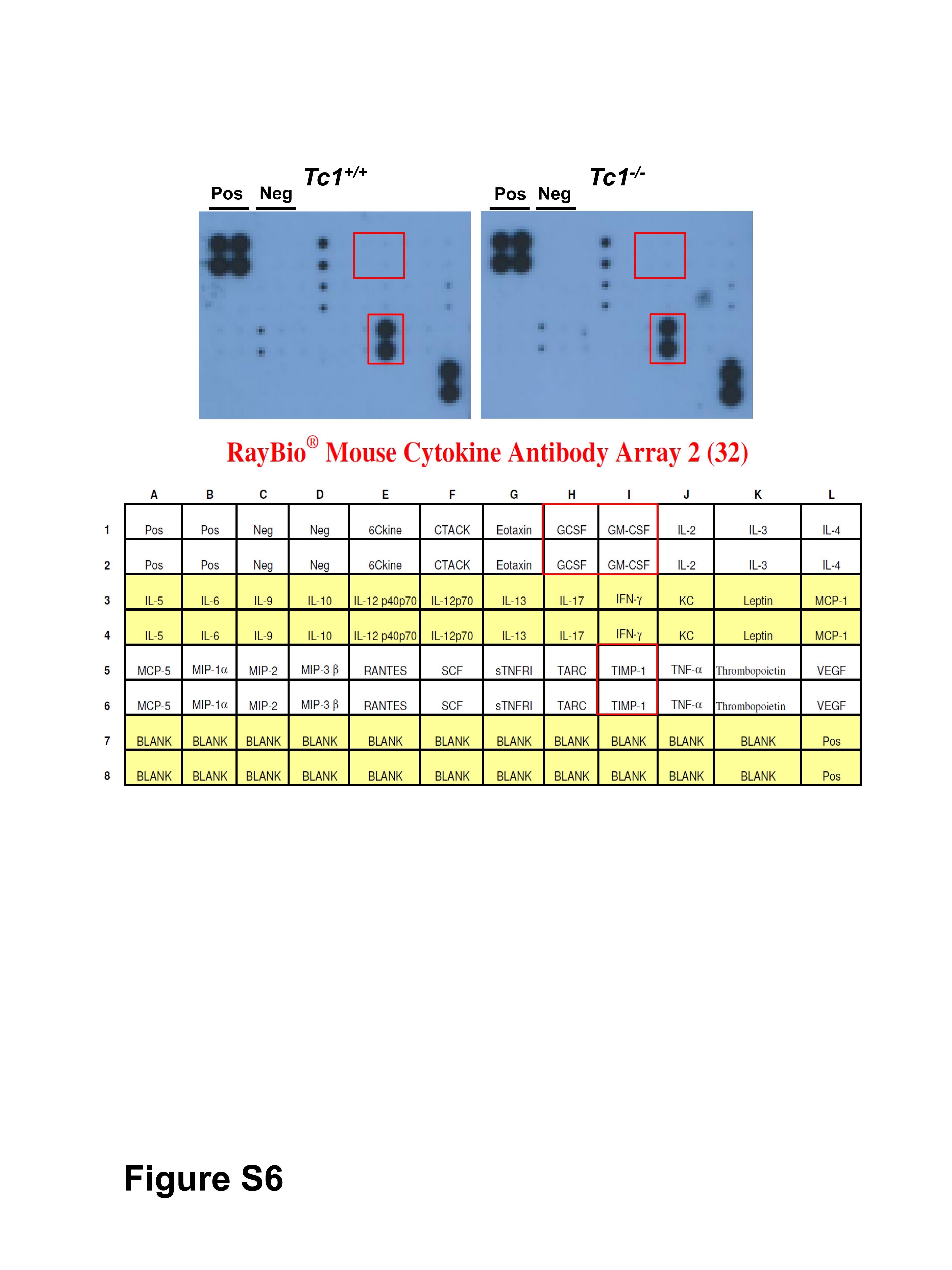

Supplement: Figure S6 — Mouse cytokine array assay using bone marrow flushed serum-free media from Tc1 −/− and wild type mice. The positions for G-CSF/GM-CSF (upper) and TIMP-1 (lower) are indicated in red squares. Positive and negative controls are indicated. (TIF) [file pone.0100311.s006.tif]

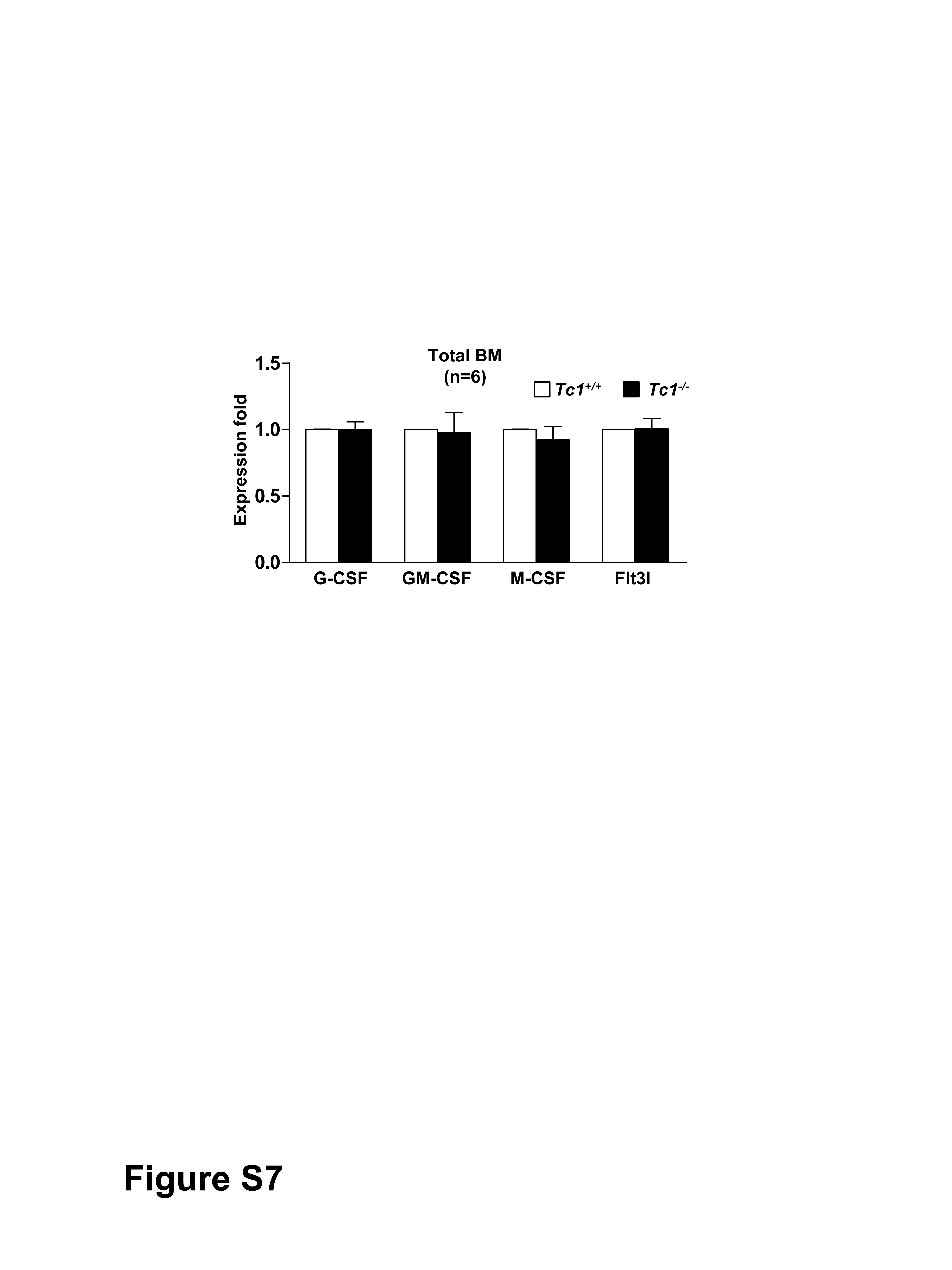

Supplement: Figure S7 — qPCR analysis of total bone marrow cells for G-CSF, GM-CSF, M-CSF, and Flt3l. Data represent mean ± s.d. of 6 male, 9 week-old Tc1 −/− mice, and 6 sex- and age-matched control mice over 3 independent experiments. (TIF) [file pone.0100311.s007.tif]
